# An Efficient Algorithm for Computing Attractors of Synchronous And Asynchronous Boolean Networks

**DOI:** 10.1371/journal.pone.0060593

**Published:** 2013-04-09

**Authors:** Desheng Zheng, Guowu Yang, Xiaoyu Li, Zhicai Wang, Feng Liu, Lei He

**Affiliations:** 1 School of Computer Science and Engineering, University of Electronic Science and Technology of China, Chengdu, Sichuan, China; 2 Departmnent of Electronic Engineering, University of California Los Angeles, Los Angeles, California, United States of America; 3 Department of Pathology and Laboratory Medicine, David Geffen University of Califonia Los Angeles School of Medicine, Los Angeles, California, United States of America; Rutgers University, United States of America

## Abstract

Biological networks, such as genetic regulatory networks, often contain positive and negative feedback loops that settle down to dynamically stable patterns. Identifying these patterns, the so-called attractors, can provide important insights for biologists to understand the molecular mechanisms underlying many coordinated cellular processes such as cellular division, differentiation, and homeostasis. Both synchronous and asynchronous Boolean networks have been used to simulate genetic regulatory networks and identify their attractors. The common methods of computing attractors are that start with a randomly selected initial state and finish with exhaustive search of the state space of a network. However, the time complexity of these methods grows exponentially with respect to the number and length of attractors. Here, we build two algorithms to achieve the computation of attractors in synchronous and asynchronous Boolean networks. For the synchronous scenario, combing with iterative methods and reduced order binary decision diagrams (ROBDD), we propose an improved algorithm to compute attractors. For another algorithm, the attractors of synchronous Boolean networks are utilized in asynchronous Boolean translation functions to derive attractors of asynchronous scenario. The proposed algorithms are implemented in a procedure called *geneFAtt*. Compared to existing tools such as *genYsis*, *geneFAtt* is significantly  faster in computing attractors for empirical experimental systems.

**Availability:**

The software package is available at https://sites.google.com/site/desheng619/download.

## Introduction

Biological networks contribute a mathematical analysis of connections found in ecological, evolutionary, and physiological studies, such as genetic regulatory networks [1]. Pursuit for the nature of these networks is the central problem for biologists [2]–[4]. In the past decades, a wide variety of research has focused on modeling genetic regulatory networks using Boolean networks and search for their attractors [5]–[10]. Computing the attractors in the Boolean networks is critical in understanding corresponding genetic regulatory networks and coordinated cellular processes such as cell cycle and cell differentiation in living organisms [6], [11]. In classical Boolean networks (CBNs), all nodes update their values at the same time called as synchronous Boolean network (SBNs). However, a criticism of CBNs as models of genetic regulatory networks is that genes do not update their values all simultaneously. To reflect this property of gene regulatory networks, *Harvey* and *Bossomaier* defined asynchronous Boolean networks (ABNs) where the random nodes were selected at each time and updated [12]. Since that, depending on the different update schemes, Boolean networks can be generally categorized into synchronous Boolean networks [7]–[9] and asynchronous Boolean networks [7], [10], [13], [14]. For the same update schemes with different priority of activator or inhibitor in genetic regulatory networks, classical equations [15], prior inhibitor equations [10] and a combination of these two [16] are three types of Boolean translation functions. Therefore, given a Boolean network, there will be 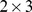 different methods to represent its Boolean translation function.

In a synchronous Boolean network, all genes update their values simultaneously at consecutive time points. *Heidel et al.* [17] and *Farrow et al.* [18] have proposed a scalar equation approach to compute attractors in SBNs. Based on the former, *Zhao* [19] has proven that the way of computing attractors in SBNs is a NP-complete problem. *Dubrova et al.* [20] have presented two tools - *BooleNet* and *Bns* - to compute attractors of SBNs. By contrast, in an asynchronous Boolean network, all genes update their values at different time points. Because each interaction between two nodes of a biological network follows distinct kinetics, it is generally thought that ABNs more realistically represent biological networks. However, due to the complexity of ABNs, the algorithms for computing network attractors are still mostly based on SBNs.

**Figure 1:**
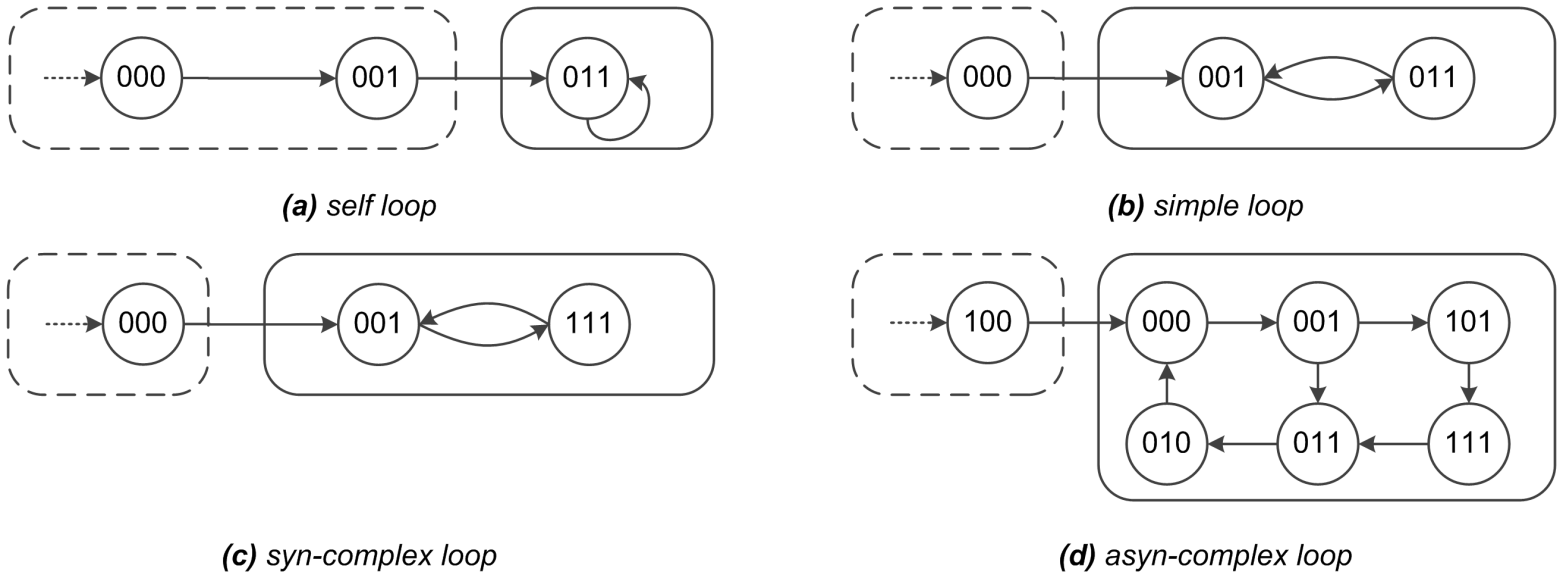
Attractors in Synchronous/Asynchronous Boolean Networks. Figure 1. Diagrams of four types of attractors in Boolean networks. Attractors are outlined by slide boxes, and transient states by dashed boxes. *(a)* A *self loop* is a single state attractor. *(b)* A *simple loop* includes two or more states: each state is connected with only another state, and any two adjacent states differ from each other by only one bit. *(c)* A *syn-complex loop* is similar to *simple loop*, but any two adjacent states differ from each other by more than one bit. *(d)* A *asyn-complex loop* includes multiple interlinked states: each state is connected with more than one states, and there is only one bit difference between any two adjacent states. In Boolean networks, the *self loop* and *simple loop* can be identified in both synchronous Boolean networks and asynchronous Boolean networks. But the *syn-complex loop* only exists in the synchronous Boolean networks, and the *asyn-complex loop* only exists in asynchronous Boolean networks.

Previously, *Garg et al.* proposed a solution to compute the attractors in both SBNs and one class of ABNs [10]. First, they demonstrated that there were four types of attractors in a Boolean network: *self loop*, *simple loop*, *syn-complex loop [or simple loop (type2)]*, and *asyn-complex loop*, shown as Fig. 1. The first two types (i.e. *self loop* and *simple loop*) were shared by SBNs and ABNs. But the latter two types, the *syn-complex loop* and the *asyn-complex loop*, were respectively found in SBNs and ABNs. Subsequently, they developed a series of algorithms which could be applied to compute the four types of attractors in a given Boolean network. Based on Garg’s contribution, *Ay F et al.* [16] gave a faster method to list the states of *self loops* and *one outgoing edge*. Both *Garg et al.* and *Ay et al.* used the *ROBDD* data structure to support their algorithms.

Here, we developed two algorithms to further improve the computation of complex attractors in both SBNs and ABNs. First, based on the works of *Garg et al.*, and *Ay et al.*, we show that iterative computing can be used to accelerate the identification of the attractors of SBNs. Second, we develop a method to compute the *asyn-complex loop (complex loop)* using *syn-complex loop*, which allows us to simplify the computation of attractors of complex loops in ABNs. We have a software package to accomplish our two algorithms which are used to locate attractors of Boolean dynamic networks (for both SBNs and ABNs), with significantly reduced time. The structure of this paper is organized as follows: Section 2 gives the methods to compute attractors and splits them into two subsections. Section 2.1 presents iterative computing attractors’ theory and its algorithms for SBNs. Section 2.2 proves a novel algorithm to locate attractors of ABNs from attractors of SBNs by asynchronous Boolean translation functions (ABTF). Section 3 tests feasibility and efficiency of our algorithm by several classical experimental benchmarks. Section 4 gives a conclusion and description of the future work.

## Methods

This section gives two methods to compute attractors in both SBNs and ABNs. The first subsection presents iterative computing attractors’ theory and its algorithms for SBNs. The second subsection provides a novel algorithm to locate attractors of ABNs from attractors of SBNs by asynchronous Boolean translation functions.

### Computing Attractors in Synchronous Boolean Networks

In a synchronous Boolean network, all nodes update their values simultaneously at consecutive time points [18]. In another word, at a given time 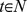, each node has only one Boolean value: 1 (Active) or 0 (Inhibit) [19]. Then, the equation of a synchronous Boolean network with 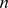 nodes is shown as Eq. 1 [19].
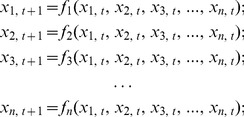
(1)where 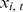 is a node in SBNs, 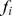 represents the Boolean function of node 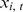, 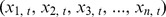 is a state in 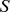, 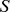 denotes the universal set with 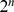 different states, 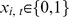, 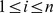. It can be simplified as follows.
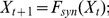
(2)where 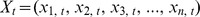, 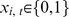, 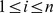, 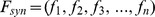 is the synchronous Boolean translation function (SBTF) from 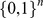 to 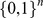. 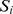 is a subset of 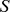. 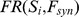 is a set of forward reachable states, which are all the states that can be reached from the states set 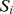 by 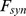. 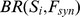 is a set of backward reachable states, which are all the states that can reach the states set 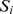 by 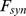. All the states in 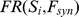 or 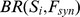 are different.

#### Definition 1

An Attractor [10]: It is the set of states 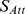 *such that for all the states* 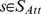, *their* 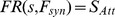, *shown as Fig. 1 (a),(b) and (c) in solid line boxes.* 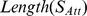 *represents state number of attractor* 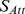. 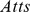 *is the union set of all the different attractors* 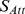 *, that is,* 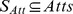.

#### Remark 1

*Definition 1 defines an attractor of a synchronous Boolean network. So similarly, we can define an attractor of an asynchronous Boolean network, when using* 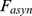 *instead of* 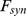 *, shown as Fig. 1(a),(b) and (d) in solid line boxes.* 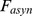 *represents an asynchronous Boolean translation function which will be introduced in section. We also use* 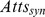 *and* 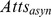 *to represent all the attractors of a synchronous Boolean network and its asynchronous Boolean network, respectively. If a state* 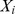 *is in an attractor,* 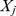 *is one of its transient states, where* 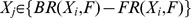, 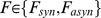 *, shown as Fig. 1 in dotted line boxes.*

Because an attractor of a Boolean network is not known in advance, a common way to address this problem is setting a randomly chosen state as the initial state and exhaustively searching the entire state space. This approach has been successfully applied in several studies to compute the network attractors using empirically derived biological networks. However, the computational burden of this approach increases exponentially with respect to the number and length of attractors. Thus, it limits the application of this method for large biological networks.

Due to the recurrent nature of attractors, we reason that iterative computing algorithms can be applied on the Boolean translation functions of SBNs, like 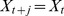. An important implication is that identifying all attractors (Definition 1) does not require the computation of the entire state space. This suggests that we can use iterative computing to accelerate the identification of attractors in a given Boolean network. In the following, we present three theorems and their proof for iterative computation. Incorporating these theorems, an algorithm is demonstrated to compute attractors of SBNs.

#### Theorems of computing attractors using iterative computing in synchronous boolean networks

According to Eq. 2, it is easily inferred that 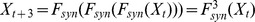, where 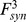 is synchronous Boolean translation function 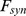 after iteratively computing 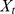 three times. Therefore, a simplified form of iterative computational equations is described as below.
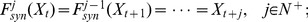
(3)where 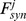 is 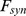 after 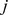 times iterative computing. 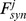 can compute the state 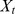 to state 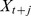 directly instead of 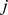 iterative computing steps by 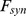. If state 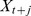 is same with state 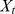, that means state 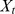 is in an attractor, which can be located as much as 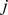 steps iterative computation.

#### Definition 2


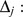
 *it represents the states that will return to themselves after* 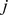 *iterations, where* 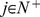 *. This can be described by Eq. 4.*

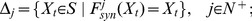
(4)


Definition 2 gives a simplified description of attractors, whose states could return to themselves after finite iterations. A shallow example can be supposed that, in a synchronous Boolean network, there are two attractors with length of 1 and 3 respectively. 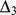 is the sum of the two attractors. Because the attractor whose length is 1 could also return to itself after 3 iterations. This feature can be proved by Theorem 1.

#### Theorem 1

*For all 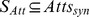, if 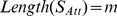, 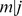 (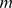 is a factor of 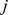), then, 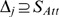.*

##### Proof

Let 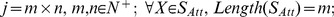


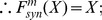


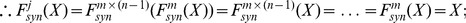


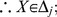


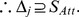



According to Theorem 1, a set of attractors, whose length is 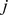, can be located after 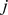 steps of iterative computing, shown as Eq. 5.
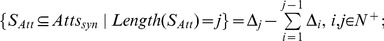
(5)


If a synchronous Boolean translation function 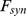 is same with 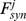, that means all the states can return to themselves by less than 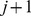 iterations. This is an important character to identify the numerous attractors in the SBNs, which has been proved by Theorem 2 and 3.

#### Theorem 2

*Given a synchronous Boolean translation function 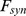 with 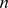 nodes, after 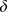 iterations, if 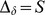, the period of this synchronous Boolean network is 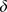, where 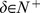.*

##### Proof

*We need to prove* 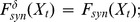


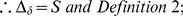


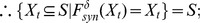


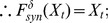


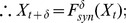


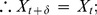


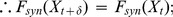


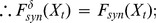




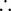
 *The period of this synchronous Boolean network is* 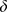.

#### Theorem 3

*Given a synchronous translation function 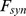 with 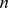 nodes, after 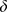 iterations, if 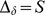, all the states are in attractors.*

##### Proof



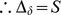


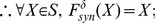


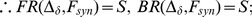


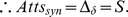



#### An improved algorithm to compute attractors in synchronous boolean networks

Combining iterative computing (Theorem 1, 2, 3 and Eq. 5) and the ROBDD data structure, we have developed Algorithm 1 to compute attractors in SBNs. The input of Algorithm 1 is the synchronous Boolean translation function 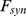; its output is states of attractors (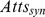) and number of all attractors (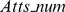). Specifically, Algorithm 1 starts with an initializing part, which initializes all the necessary variables. This is followed by a resolving part, which computes and deletes redundant attractors in a network. The resolving part further contains four components. The first component (Lines 12–13) will continue the next iterative computing and delete the visited attractors based on Theorem 1 and Eq. 5. The second component (Lines 15–20) judges whether synchronous Boolean translation functions are periodic or not, which has been proved by Theorem 2 and 3. The third component (Lines 22–27) verifies whether there will be a new attractor generated after one iteration. That is, if existing a new attractor, Algorithm 1 will add it into attractors (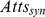). Meanwhile, it will also update the attractors’ number (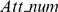) and continue the next iteration. If not, Algorithm 1 will go to the next iterative computing. The last component (Lines 11, 29) contains the fix-point condition. When satisfying this condition, it will output attractors and number of attractors. For more detailed information, please read the Algorithms 1.

In the initializing part, 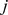 is the times of iterations. 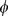 is an empty set. 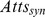 and 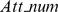 are the attractors and number of attractors, respectively. 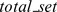 is the fix-point condition to judge whether the algorithm can be terminated or not. 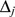 represents the states that will return to themselves after 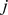 iterations by synchronous Boolean translation function 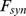. 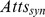, 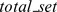 and 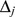 are ROBDD data structures. In the main resolving part, 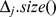 represents state number of 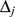. 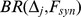 are all the states that can reach to 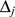 by synchronous Boolean translation function 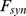.

Algorithm 1 is different with *Garg et al.*, which randomly picks up a state from state space and computes its forward reachable states to get an attractor. If you want to find out the attractors whose length is 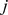, it needs to exhaustively search the state space. However, our algorithm can easily compute the same attractors in 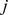 times iterative computing.

**Algorithm 1: Iterative Computing Attractors on Synchronous Boolean Translation Function**

**Function:** 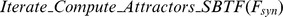 will compute all the attractors of SBTF iteratively;

**Input:** The synchronous Boolean translation function 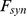;

**Output:** All attractors (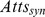) and number of attractors (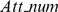);

**1 begin**

**2**//Initializing part

**3** **begin**

**4** 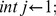 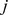 is times of iteration

**5** 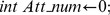 //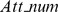 is the number of attractors

**6** 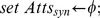 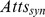 is a set of attractors

**7** 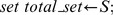 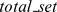 is a set of unvisited states

**8** 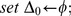 Initialize 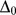 as empty set

**9** **end**

**10** //Main resolving part

**11** **while** 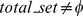 **do**

**12** 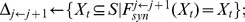
One iterative computing

**13** 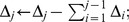 //Delete the visited attractors as Theorem 1 & Eq. 5

**14**    //This part is equivalence with Theorem 2 and 3

**15    if
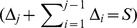
then**

**16**       report attractors 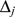 and its number 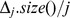

**17** 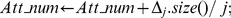 //Update the value of

**18** 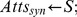 //All states are in attractors by Theorem 2 and 3

**19** 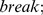 Exit the while loop

**20    end**

**21**      //If existing unvisited attractors, record them

**22    if
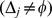
** **then**

**23**      report attractors 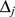 and its number 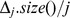

**24** 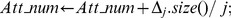 //Update the value of 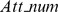

**25** 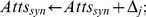 //Add the new attractors into 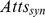

**26** 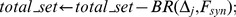 //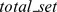 deletes the states can reach 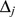

**27** **end.**

**28 end.**

**29 return** 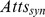, 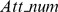;

**30 end.**

### Computing Attractors in Asynchronous Boolean Networks

As mentioned earlier, SBNs and ABNs differ in nodes updating schemes of Boolean translation functions. Instead of updating values of all the nodes simultaneously, ABNs only allow some of the nodes to update their values at a time point. For this reason, the computing of attractors in ABNs is more time consuming. Especially, it needs more intermediate steps when there are more than one bit different between two states.

#### Analysis of attractors in asynchronous boolean networks

It is essential to give a simple description of types of attractors. As represented in Fig. 1, there are four types of attractors, *self loop*, *simple loop*, *syn-complex loop* and *asyn-complex loop* in both SBNs and ABNs. A *self loop* is a single state attractor, shown in Fig.1 (a). A *simple loop* includes two or more states, where every state is connected with only another state, and any two adjacent states differ from each other by only one bit, shown in Fig.1 (b). A *syn-complex loop* is similar to *simple loop*, but any two adjacent states differ from each other by more than one bit, shown in Fig.1 (c). A *asyn-complex loop* includes multiple interlinked states: every state is connected with more than one states, and there is only one bit different between any two adjacent states, shown in Fig.1 (d). Fig. 1 (a)(b)(c) and Fig. 1 (a)(b)(d) stand for the different types of attractors in SBNs and ABNs, respectively.

According to the properties of *self loops* and *simple loops*, they can easily be identified in SBNs, which also are same in ABNs. Interestingly, a closer examination of the structure of the *syn-complex loops* and *asyn-complex loops* suggests that every *asyn-complex loop* contains one *syn-complex loop* or some *transient states*. This suggests that it is possible to use *syn-complex loop* to easily locate the states in *asyn-complex_loop* by asynchronous Boolean translation functions.

**Figure 2:**
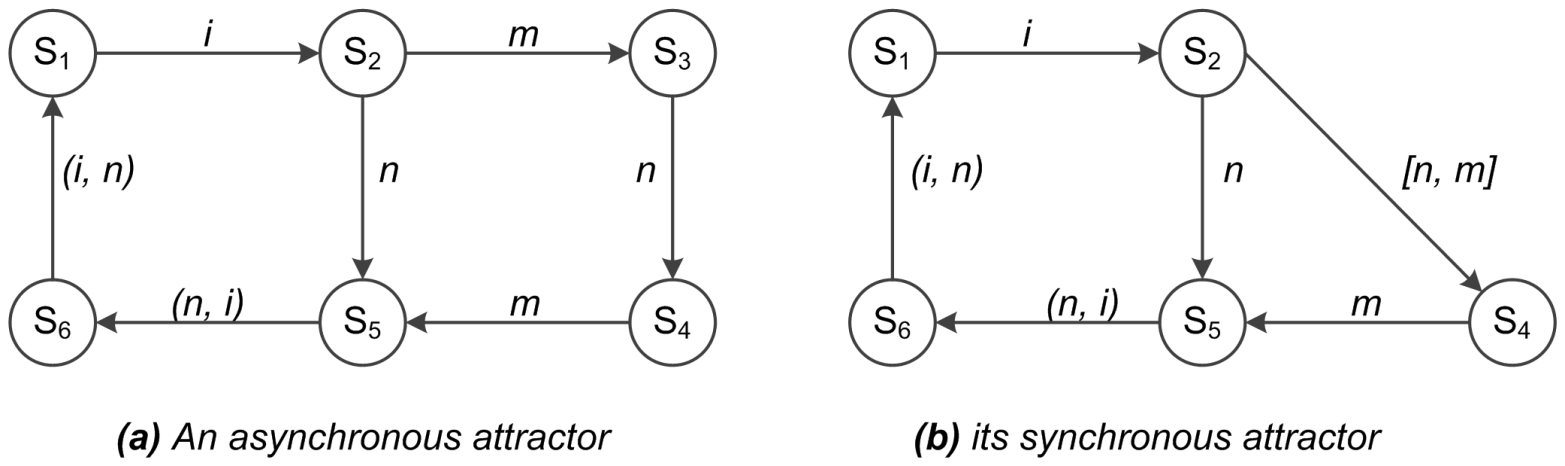
An Asynchronous Attractor to Synchronous Attractor. Figure 2. Diagrams of an attractor in asynchronous (a) and synchronous (b) Boolean networks. Each state is represented by a circle, and is designated as 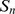. The variable 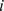 represents that the 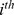 bit of the state 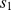 and 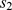 is different, which is also same as 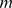 and 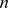. The numbers 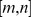 indicate that state 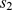 and 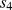 differ by the 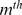 and 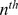 bits respectively. The 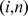 and 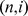 represents when state 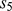 and 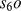 differ at the 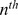 bit, state 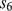 and 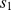 will be different at the 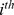 bit, and vice versa. The difference between the two representations (i.e. synchronous versus asynchronous) of the attractor is that 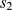 and 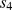 differ in the 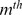 and 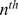 bits, 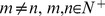. That means we can use *syn-complex loop* to easily locate the states in *asyn-complex_loop* by asynchronous Boolean translation function 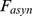.

One example is shown in Fig. 2, here 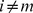, 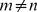, 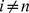, 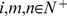. Fig. 2(a) is an asynchronous attractor, where the current state and its next states are different by one bit. Suppose that the 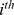 bit of the state 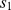 and 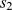 is different. If 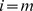, it means that state 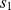 and 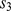 are the same. The situation is also true for 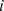 and 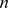. If state 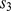 and 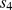 are different at the 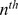 bit, then state 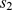 and 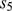 must differ at the 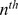 bit. Otherwise, state 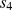 cannot reach state 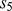 by changing one bit. When state 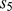 and 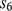 differ at the 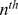 bit, state 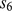 and 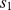 will be different at the 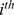 bit, and vice versa. Fig. 2(b) shows the corresponding synchronous attractor to Fig. 2(a). The difference is that state 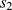 and 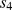 differ in the 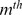 and bits simultaneously. Other relations are the same except for state 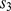. Therefore, an *asyn-complex_loop* contains one *syn-complex loop* or some *transient states*. That means we can use *syn-complex loop* to easily locate the states in *asyn-complex_loop* by asynchronous Boolean translation function 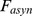.

#### An algorithm to compute attractors in asynchronous boolean networks

An important implication of the above analysis is that the attractors of the ABNs can be derived from the attractors of the SBNs using synchronous and asynchronous Boolean translation functions. Therefore, we use attractors computed by Algorithm 1 in SBNs as the basis input for the new algorithm (Algorithm 2) to compute attractors of ABNs.

Specifically, Algorithm 2 can be divided into three parts: initializing part (Lines 3–6), main resolving part (Lines 8–41) and checking unvisited states part (Lines 44–52). The initializing part also initializes all necessary variables in Algorithm 2. The main resolving part also can be split into five components. The first component (Lines 11–15) means 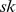 is a *self loop*. The second component (Lines 18–21) means 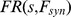 is a *simple loop*. The third component (Lines 24–29) means 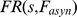 is an unvisited *asyn-complex loop* and 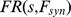 is an unvisited *syn-complex loop*. The forth component (Lines 32–34) means 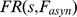 is a visited *asyn-complex loop* and 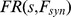 is an unvisited *syn-complex loop*. The fifth component (Lines 37–40) means 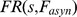 is the set of *transient states* and 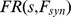 is an unvisited *syn-complex loop*. After the main resolving part, if there exists unvisited states, Algorithm 2 will go to the checking unvisited states part. This part will check the unvisited states and report the left *asyn-complex loops*. For more detailed information, please read the Algorithms 2.

Furthermore, in the initializing part, 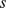 is a state, 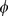 is an empty set, 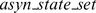 is a set of recording unvisited states by 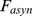, 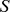 is the universal set. In the main resolving part and checking unvisited part, 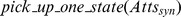 represents picking up anyone state from attractors 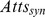 of SBNs. 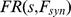 and 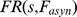 are the reachable states from 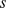 by synchronous Boolean translation function 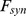 and asynchronous Boolean translation function 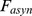, respectively. 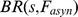 is the reachable states to 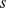 by asynchronous Boolean translation function 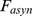. We note that, our approach of identifying attractors is totaly different with *Gary et al.* and *Ay et al*. It is more efficient to compute the *asyn-complex loop* because it can easily locate the states in 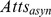.

**Algorithm 2: Compute and classify Four Types Attractors**

**Function.** 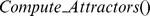 computes and classfies attractors as four types, shown in Fig. 1;

**Input.** Attractors of SBN(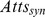), SBTF(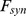) and ABTF(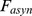);

**Output.** Four types attractors (a)(b)(c)(d) of SBTF and ABTF shown as Fig. 1;

**1** **begin**

**2**      // Initializing part

**3** **begin**

**4** 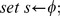 // 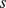 is a state

**5** 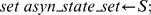 / 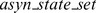 is a set of unvisited states by 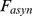

**6** **end**

**7**        // Main resolving part

**8** **while** 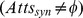 **do**

**9** 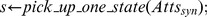 // 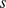 is any one state in 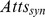

**10**        // 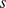 is a self loop, shown as Fig. 1(a)

**11** **if** (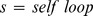) **then**

**12** *report s is a self loop state*

**13** 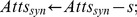 // Delete 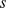 from 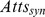

**14** 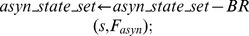 // Delete reachable states to 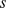

**15       end**

**16        // 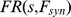
** is a simple loop, shown as Fig. 1(b)

**17       eles if** (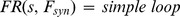) **then**

**18** *report FR(s, 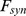) is the simple loop* **then**

**19** 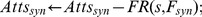 // Delete the simple loop from 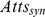

**20** 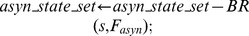//Delete reachable states to 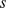

**21       end**

**22**        // 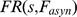 is an unvisited asyn-complex loop, shown as Fig. 1(d)

**23** **eles if** 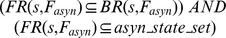 **then**

**24** 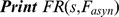 *is a asyn-complex loop*

**25**          // 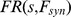 is a syn-complex loop, shown as Fig. 1(c)

**26** 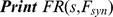 *is a syn-complex loop*

**27** 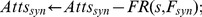 // Delete the syn-complex loop from 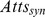

**28** 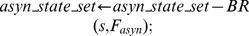// Delete reachable states to 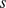

**29       end**

**30**       // 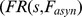 is an visited asyn-complex loop

**31** **eles if** 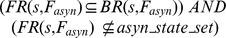 **then**

**32** 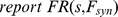 *is a syn-complex loop*

**33** 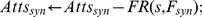 // Delete the syn-complex loop from 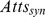

**34** **end**

**35**       // 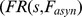 are the transient states

**36       else**

**37** 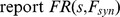 *is a syn-complex loop*

**38** 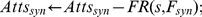 // Delete the syn-complex loop from 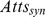

**39** 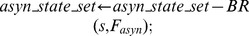// Delete reachable states to 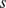

**40** **end**

**41** **end**

**42**     // Checking unvisited states

**43** **while** 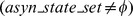 **do**

**44** 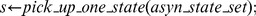 // 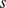 is a state in 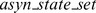

**45** **if** 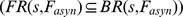 **then**

**46** 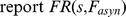 *is a asyn-complex loop;*

**47** 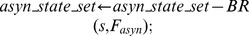// Delete reachable states to 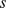

**48** **end.**

**49** **else.**

**50** 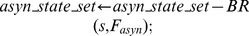// Delete reachable states to 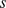.

**51** **end.**

**52    end.**

**53 end.**

## Results and Discussion

We have implemented our methodology in a software package called *geneFAtt*, which is based on a ROBDD data structure named BuDDy [21]. In this package, there are two parts, source code and benchmark. The source code part implements functions of Algorithm 1 (iterative computing attractors in synchronous Boolean networks) and Algorithm 2 (computing and classifying attractors as four types of synchronous Boolean networks and asynchronous Boolean networks). Algorithm 1 computes the attractors of SBNs, which are then used as the input of Algorithm 2 to classify the attractors of ABNs. Because the *self loop* (Fig. 1(a)) and *simple loop* (Fig. 1(b)) of SBNs are same with its corresponding type of attractors of ABNs, respectively. We can easily use *syn-complex loops* (Fig. 1(c)) to locate the *asyn-complex loops* (Fig. 1(d)) by the asynchronous Boolean translation functions. The benchmark part includes five biological networks, *Mammalian Cell* [7], *T-helper* [22], *Dendritic Cell* [10], *T-cell Receptor* [23], and *Protein-ex* [17]. We ran *geneFAtt* and *genYsis* [10] with the five synchronous/asynchronous biological models and have compared their running results.

As shown in Table 1, the first four biological networks, *Mammalian Cell* [7], *T-helper* [22], *Dendritic Cell* [10] and *T-cell Receptor* [23] have been studied in [10]. *Protein-ex* is an extended case from *Heidel et al.* [17]. It also represents a kind of biological networks that all the states are in attractors. Because we adopt to the same methods of modeling the SBNs and ABNs to setup the synchronous and asynchronous Boolean translation functions refereed to *Garg et al.* Meanwhile, using the same inputs, *geneFAtt* can obtain the same attractors as those by *genYsis* [10] shown in Table 1, which suggests that our algorithms are valid for the analysis of biological networks.

**Table 1:** Characters of Five Different Biological Networks.

Benchmark	Attractors’ Number			
	Self Loop	Simple Loop	Syn-complex Loop	Asyn-complex Loop
Mammalian Cell	1	0	1	1
T-helper	3	0	0	0
Dendritic Cell	0	1	0	0
T-cell Receptor	1	0	9	7
Protein-ex	2	0	4114	0

With the same validity, *geneFAtt* shows more efficient and feasible compared to *genYsis*. Table 2 gives the running time and running time efficiency ratio (RTER, Eq. 6) of the five biological networks which have been produced by the two procedures *genYsis* and *geneFAtt*. 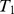 and 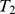 represent the running time on every biological network of the two procedures *genYsis* and *geneFAtt*, respectively. All experiments are performed on an Intel® Core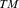 CPU 4300 1.80 GHz with 2GB memory and a Ubuntu 9.04 Linux server. Importantly, we note that the running time of *geneFAtt* is much more shorter than *genYsis* [10]. Specifically, compared with *genYsis*, *geneFAtt* improves the running time of *Mammalian Cell* [7], *T-helper* [22], *Dendritic Cell* [10], *T-cell Receptor* [23], and *Protein-ex* [17] by *3.25, 8.19, 116.00, 23.48, and 77.05* times, respectively. Remarkable, *geneFAtt* improves the running time of the Dendritic Cell ([10]) gene network by a striking *116.00* times.
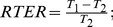
(6)


**Table 2:** Performance Comparison between genYsis [10] and geneFAtt.

Benchmark	Time (*sec*)		RTER
	genYsis [10]	geneFAtt	
Mammalian Cell	0.102	0.024	3.25×
T-helper	0.193	0.021	8.19×
Dendritic Cell	0.351	0.003	116.00×
T-cell Receptor	330.643	13.506	23.48×
Protein-ex	86.162	1.104	77.05×

### Conclusions

This paper has addressed a method to compute attractors in SBNs and ABNs. We have developed a new iterative computing algorithm to identify the attractors 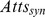 of SBNs. Meanwhile, another computing and classifying algorithm has been proposed to locate the attractors of ABNs rapidly. Our approaches give a significant acceleration of computing and locating attractors in biological networks. It is a big challenge that how to identify attractors in the large biological networks. Based on above research, it is expected that we can find a pathway to resolve this problem from the modeling methods of biological networks.
